# CT-on-Rails Versus In-Room CBCT for Online Daily Adaptive Proton Therapy of Head-and-Neck Cancers

**DOI:** 10.3390/cancers13235991

**Published:** 2021-11-28

**Authors:** Konrad P. Nesteruk, Mislav Bobić, Arthur Lalonde, Brian A. Winey, Antony J. Lomax, Harald Paganetti

**Affiliations:** 1 Department of Radiation Oncology, Massachusetts General Hospital and Harvard Medical School, Boston, MA 02114, USA; mbobic@mgh.harvard.edu (M.B.); alalonde@mgh.harvard.edu (A.L.); winey.brian@mgh.harvard.edu (B.A.W.); hpaganetti@mgh.harvard.edu (H.P.); 2 Department of Physics, ETH Zurich, CH-8093 Zurich, Switzerland; tony.lomax@psi.ch; 3 Center for Proton Therapy, Paul Scherrer Institute, CH-5232 Villigen, Switzerland

**Keywords:** adaptive proton therapy, CT-on-rails, CBCT, Monte Carlo, head-and-neck cancers, positioning uncertainties

## Abstract

**Simple Summary:**

Daily adaptive proton therapy will allow the unique properties of protons to be fully exploited. Cone-beam CT (CBCT) is the primary imaging modality considered for daily adaptation due to its low cost, compactness, and thus wide availability. However, there are proton therapy centers equipped with CT-on-rails or considering the installation of such scanners for the so-called “near-treatment-position” imaging. Our study addresses the critical question, whether CT-on-rails is a suitable modality for daily adaptive proton therapy. Although high precision accuracies have been claimed for CT-on-rails, no quantitative study of the adaptation efficacy with increased treatment execution uncertainties has ever been performed. In this paper, we demonstrate that the expected uncertainties will not affect the dosimetric efficacy of the adaptation based on in-room CT for head and neck cancers, and thus CT-on-rails applied to “near-treatment-position” imaging is a suitable modality for online adaptive proton therapy.

**Abstract:**

Purpose: To compare the efficacy of CT-on-rails versus in-room CBCT for daily adaptive proton therapy. Methods: We analyzed a cohort of ten head-and-neck patients with daily CBCT and corresponding virtual CT images. The necessity of moving the patient after a CT scan is the most significant difference in the adaptation workflow, leading to an increased treatment execution uncertainty *σ*. It is a combination of the isocenter-matching *σ_i_* and random patient movements induced by the couch motion *σ_m_*. The former is assumed to never exceed 1 mm. For the latter, we studied three different scenarios with *σ_m_* = 1, 2, and 3 mm. Accordingly, to mimic the adaptation workflow with CT-on-rails, we introduced random offsets after Monte-Carlo-based adaptation but before delivery of the adapted plan. Results: There were no significant differences in accumulated dose-volume histograms and dose distributions for *σ_m_* = 1 and 2 mm. Offsets with *σ_m_* = 3 mm resulted in underdosage to CTV and hot spots of considerable volume. Conclusion: Since *σ_m_* typically does not exceed 2 mm for in-room CT, there is no clinically significant dosimetric difference between the two modalities for online adaptive therapy of head-and-neck patients. Therefore, in-room CT-on-rails can be considered a good alternative to CBCT for adaptive proton therapy.

## 1. Introduction

Proton therapy with pencil-beam scanning has capabilities to deliver highly conformal dose distributions by optimizing both the positions and energies of many small proton beamlets. However, it is still challenging to fully exploit these capabilities clinically. One of the biggest challenges is daily variations in patient’s geometry due to the patient setup and interfraction anatomy changes. The latter can be due to the patient’s weight variation, tumor progression or shrinkage during the treatment, or sinus filling in the case of head and neck tumors. The resulting uncertainties might compromise the planned target coverage and dose constraints for organs at risk (OAR). One of the most promising solutions is adaptive treatment when the original plan is adapted to daily variations in patient positioning and anatomical changes. Such an approach using various imaging devices has been extensively studied specifically for head and neck cancers in conventional photon therapy [1,2,3,4,5,6,7]. However, adaptive proton therapy represents unique challenges (range uncertainties) and capabilities (high conformality). The precise knowledge of the range of the delivered beamlets requires accurate proton stopping powers to be derived from daily patient images. Adaptive approaches with protons will also be more sensitive to dose calculation uncertainties.

Two conceptually different daily adaptation approaches have been suggested. One adaptation workflow is based on fast GPU Monte Carlo dose calculation and Cone-Beam CT (CBCT) imaging [8,9]. Alternatively, an approach based on in-room CT imaging and analytical dose recalculation has been proposed [10,11,12,13]. In terms of different dose calculation methods, analytical computations are faster, but Monte Carlo dose calculation has been shown to increase accuracy in proton therapy significantly [14]. For instance, in head and neck cancers, analytical dose calculation accuracy is compromised due to tissue heterogeneities [15,16,17,18]. Another difference between the two adaptation approaches is the imaging modality. The choice of the imaging modality is often dictated by its availability. Due to the lower costs and compactness, the worldwide availability of CBCT in proton centers is larger. However, some facilities consider installing an in-room CT scanner for so-called “near-treatment-position” imaging.

Using CT-on-rails has several advantages, most importantly the higher image quality (Table 1). It makes the comparison of the daily and reference planning CT image much easier and less prone to uncertainties. CT images are also much more suitable than CBCT for contouring by a physician. In terms of adaptive approaches, it may be advantageous in case of significant interfraction anatomical changes when deforming original contours is not effective anymore. Then, online target delineation for plan adaptation may be performed. For instance, such an approach of fast replanning based on daily CT taken with in-room CT-on-rails has been reported for photon treatments of the prostate [19,20]. In terms of the extra whole-body dose to the patient over the course of the treatment, CT-on-rails might give slightly lower dose, as shown in Table 1. Moreover, adaptation with CT-on-rails does not exclude the use of low-dose scanning protocols, as with this modality, even lower currents lead to acceptable image quality. However, it must be noted that CBCT technology is quickly progressing, and, depending on the particular scanner and protocol used, whole-body doses can be very different and potentially equal to doses for fan-beam CT. It is also not easy to evaluate and report imaging doses and different methodologies are used [21]. Another advantage of CT-on-rails is the use of the same modality for both planning and daily adaptation. Furthermore, dose calculation accuracy depends on image quality. However, with a proper scatter correction algorithm [22,23,24,25,26], the influence of lower image quality on the dose calculation can be minimized. In particular, it has already been shown that scatter-corrected CBCT can be used for accurate dose calculation for head-and-neck adaptive proton therapy with a range uncertainty well below 1 mm and a 2%/2 mm mean gamma pass rate of 98.9% for IMPT plans [22].

CBCT is generally streamlined into most radiotherapy workflows for patient setup. A clear advantage is that CBCT imaging is performed at the treatment position and requires much less time than a scan with CT-on-rails followed by the final positioning of the patient for the treatment. However, moving the patient from the CT on rails to the treatment position and performing the adaptive workflow can be done in parallel, reducing the overall impact on the treatment time.

Online plan adaptation is performed when the patient is already positioned on the couch and ready for dose delivery. Therefore, the primary factor which might compromise adaptive treatments based on CT-on-rails is an additional positioning uncertainty associated with the necessity of moving the patient after imaging but before dose delivery (Table 1). The influence of those uncertainties on dose distributions has not been shown neither for standard non-adaptive proton therapy treatments nor for adaptive approaches. As the latter is expected to eliminate or at least minimize treatment planning margins, any additional uncertainty introduced after adaptation may lead to comparable or even worse dose distributions than for standard non-adaptive treatments. Therefore, the goal of this paper is to assess how much the resulting increased treatment execution uncertainty will influence the dosimetric efficacy of daily adaptive proton therapy of head and neck (H&N) cancers, and hence address the question whether post-adaptation uncertainties in patient positioning outweigh other advantages of CT-on-rails.

## 2. Materials and Methods

### 2.1. Patient Cohort and Treatment Planning

For this retrospective study, we used the same patient cohort and treatment planning approach as for the work reported in [9,30]. The dataset included 10 H&N patients with tumors located in the oral cavity, oropharynx, and larynx. For each patient, the planning CT image and daily acquired CBCT images were available. The total number of analyzed fractions was 320 (31–35 per patient). Since the patients were treated with volumetric modulated arc therapy (VMAT), new intensity-modulated proton therapy (IMPT) plans were created using the original contours, approved by a physician. The plans were designed as 57 Gy (RBE) and 70 Gy (RBE) prescribed to the low-risk CTV and to the high-risk CTV, respectively. We did not apply any PTV or range uncertainty margin. Online adaptation is expected to allow all the margins to be significantly reduced, so our approach represents the best-case scenario with marginless plans. Such marginless plans are dosimetrically the most sensitive to post-adaptation positioning uncertainties. For both CTVs, the clinical objectives were defined as *D*_98_ ≥ 95% and *D*_2_ ≤ 107% of the prescribed dose, where *D*_98_ and *D*_2_ are the minimum doses to 98% and 2% of the CTV volume, respectively. We considered the following organs at risk (OARs) with the corresponding constraints: spinal cord (*D_max_* < 45 Gy), parotid glands (*D_mean_* < 26 Gy), constrictor muscles (*D_mean_* < 42 Gy), larynx (*D_mean_* < 40 Gy), and brainstem (*D_max_* < 54 Gy). 

All plans were created in Ray Station with three fields (60°, 180°, and 300°), each using a range shifter with a water equivalent thickness of 40 mm and a 30 mm minimum air gap. The *IBA Dedicated Nozzle* beam model was used for planning and dose calculations with the spot sigma in air ranging from 2.5 to 6.4 mm for the applicable nominal beam energies (between 225 MeV and 65 MeV, respectively). 

### 2.2. CT Data Preparation

**Figure 1:**
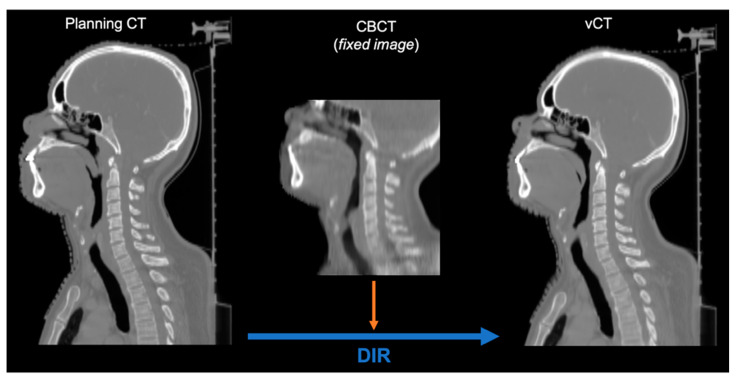
Preparation of a vCT based on a fraction-specific CBCT.

As the patient cohort contained daily CBCT images only, a corresponding dataset of virtual CT (vCT) images was prepared by means of deformable image registration (DIR). For this purpose, we applied a GPU parallelized B-spline algorithm with the mean squared error metric in Plastimatch—an open-source code for radiotherapy and imaging [31,32]. The original planning CT (moving images) was deformed to the daily CBCT (fixed image) for each fraction, as shown in Figure 1. Each fraction was assessed visually to exclude deformation artifacts that could potentially affect dose calculation. Although DIR did not always perfectly deform anatomical structures such as vertebrae, the area around the tumor volume, relevant for dose calculation, was found to be matching CBCTs for all the studied fractions. 

### 2.3. Simulation of Daily Adaptive Treatments

We performed plan adaptation with an in-house developed online framework for adaptive proton therapy [9] based on fast GPU-accelerated Monte Carlo (gPMC) calculations [33,34,35,36]. For each daily vCT, contours were propagated by performing DIR from the planning CT to the vCT in Plastimatch using the same algorithm and metric as for vCT generation. The dose was calculated on each vCT with a new deformed set of contours. Based on the resulting dose-influence matrix [8], the weights of the beamlets were adjusted using an in-house developed optimization tool initially conceived for temporo-spatial IMRT studies [37]. Such adapted plans were verified by performing dose calculations on vCTs and scoring the dose for the propagated contours.

If we neglect DIR uncertainties, the adaptation on the generated vCT dataset, as described above, is equivalent to the adaptation performed directly on CBCT images [8]. Therefore, with the above-described approach, we do not account for dose calculation uncertainties in the comparison between the two imaging modalities. As such, in terms of dosimetric efficacy, the difference between the use of CBCT vs. CT-on-rails for daily adaptive therapy is simplified to the difference in treatment execution uncertainties only. 

#### Treatment Execution Uncertainties

In the context of online adaptation, treatment execution uncertainty is the intrafraction uncertainty in patient positioning introduced after the daily imaging but before the dose delivery. Daily plan adaptation corrects only for the interfraction position and anatomic changes. Both imaging modalities are associated with some intrinsic treatment execution uncertainties, such as matching between isocenters of the imaging and beam delivery systems. However, since our goal is to compare the modalities, we assume no treatment execution uncertainty for CBCT. Therefore, uncertainties associated with the use of CT-on-rails should be considered additional uncertainties with respect to CBCT.

In the case of CT-on-rails, we have two types of additional uncertainties. The first is an extra contribution to the isocenter matching since the patient is not imaged in the treatment position, but either “near-treatment-position” imaging is performed with an in-room CT or remote imaging when a remote positioning system is used [28]. This uncertainty is dictated by the robot accuracy for in-room imaging or by the coupling system accuracy for remote positioning. In either case, it is safe to assume a conservative value of 1 mm for the isocenter matching uncertainty ***σ****_i_*_._ The second type of uncertainty ***σ****_m_* corresponds to random patient movements induced by the couch motion. It depends on the treatment site and might be patient-specific. The largest reported displacements for head-and-neck cancers correspond to remote positioning with the use of a thermoplastic mask as immobilization [28]. A standard deviation of differences in pre- and post-treatment control images was reported to be as high as 2.4 mm for two axes. Therefore, we analyze three different scenarios with ***σ****_m_*= 1, 2, and 3 mm. The total treatment execution uncertainty ***σ*** is then:

$$\sigma =\sqrt{\sigma_{i}^{2}+\sigma_{m}^{2}}$$

giving the values [***σ****_1,_**σ**_2,_**σ***_3_] = [1.41 mm, 2.24 mm, 3.16 mm] corresponding to the three scenarios. We conservatively assume equal uncertainties in each axis posterior-anterior, left-right, and inferior-superior. 

To mimic the treatments with the above-mentioned uncertainties, we introduced after daily plan adaptation a random offset Δ*x_kj_* in each axis *k =* 1,2,3 for individual fraction *j* following Gaussian distribution with ***σ*** corresponding to one of the three scenarios:

$$\Delta x_{kj}\;\sim \;N(0,\;\sigma )$$

**Figure 2:**
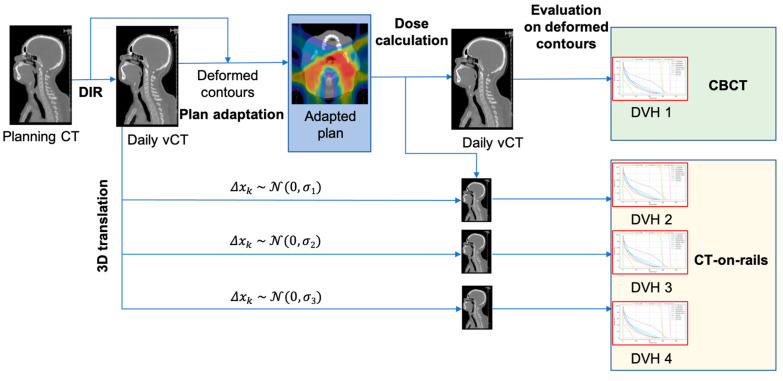
Simulation of the adaptive treatment workflow based on daily vCTs for both imaging modalities.

For each fraction *j*, vCTs were rigidly transformed with a synthetic vector field representing a uniform 3D translation [Δ*x*_1__j,_ Δ*x*_2__j,_ Δ*x*_3__j_]. Then, the dose was calculated on each transformed vCT. The simulation of the adaptive workflow with both imaging modalities is summarized in Figure 2.

### 2.4. Evaluation of Adaptation Efficacy

To compare adaption efficacy with CT-on-rails vs. CBCT, we evaluated several DVH metrics for each adaptive treatment scenario as well as for an un-adapted base plan (BP), which served as the reference. Most of the DVH metrics we used correspond to the treatment planning objectives. In terms of target volume coverage, *D*_98_ and *D*_2_ were assessed for high-risk CTV and *D*_98_ for low-risk CTV. For organs at risk, mean dose *D_mean_* was evaluated for the larynx, parotids, and constrictors. For the spinal cord, we used *D*_1cc_—the minimum dose to the most irradiated 1 cc. Apart from that, the integral dose to the healthy tissue *D* *V* was also calculated. All these metrics were evaluated for individual fractions as well as for accumulated DVHs per patient. The accumulated DVHs were obtained by registering contours back to the planning CT and warping the doses calculated on vCT for each fraction. From accumulated doses, we also evaluated discrepancies in dose distributions between CBCT and three different scenarios for CT-on-rails.

Additionally, we compared DVH metric values obtained for all three CT-on-rails scenarios with those obtained for CBCT-based online adaptation. The goal of this analysis was to check if the distributions of dosimetric values for online adaptation based on CT-on-rails are significantly different from those obtained for CBCT-based adaptation. For this purpose, we performed two-sided Mann–Whitney U test with the null hypothesis that the randomly selected DVH metric value for a given CT-on-rails scenario is equal to the randomly selected value for the CBCT-based online adaptation. The hypothesis is rejected if the *p*-value is *p* ≤ .05. 

## 3. Results

**Figure 3:**
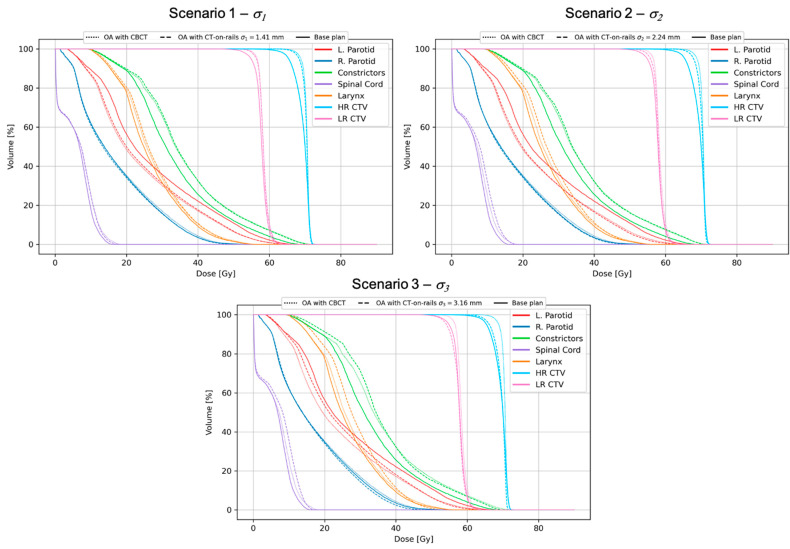
Accumulated DVHs for three offset scenarios for a chosen patient. The DVHs for the CT-on-rails offset scenarios are compared to those obtained for the unadapted base plan and CBCT-based online adaptation (OA) with no offset.

**Figure 4:**
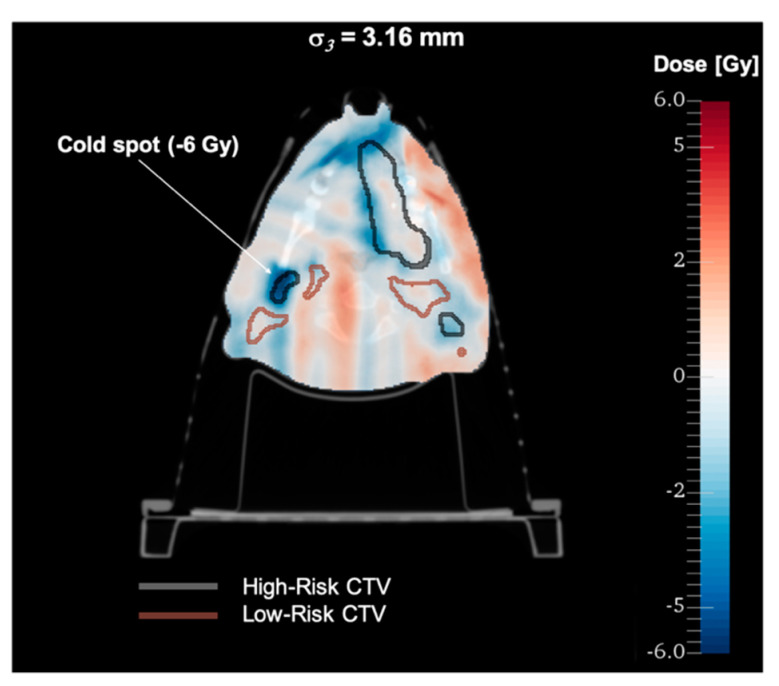
Difference in accumulated dose distribution for the largest CT-on-rails offset scenario (*σ*_3_ = 3.16 mm) with respect to CBCT-based online adaptation with no offset.

**Figure 5:**
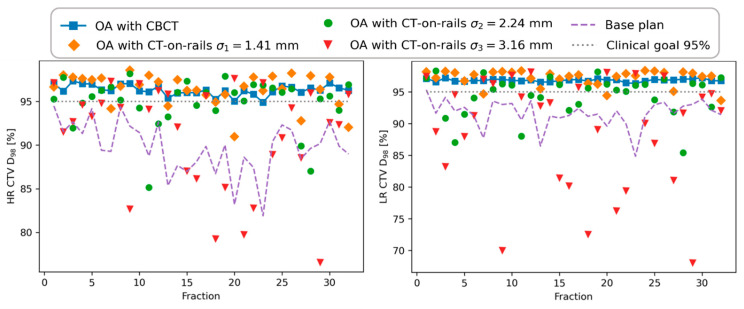
Evolution the target coverage (*D*_98_) for high-risk (HR) and low-risk (LR) CTVs and all the scenarios tested for a chosen patient.

Figure 3 shows accumulated DVHs for a 32-fraction treatment and all the offset scenarios for a patient with significant changes of the position visible in daily CBCT images. For this patient, the unadapted base plan (BP) applied to all the fractions resulted in a very poor target coverage which has been restored by online adaptation based on daily CBCT images (OA with no offset). With the introduced offsets corresponding to *σ*_1_ and *σ*_2_, there were no significant dosimetric differences observed, and the resulting DVHs and the metrics evaluated from them are clinically acceptable. Only for the largest offsets corresponding to *σ*_3_, the target coverage was compromised, being comparable to the unadapted base plan performance. This can also be seen in the dose distribution difference shown in Figure 4 for this particular scenario. Dose differences as large as 15 Gy were observed with cold spots in the target, as highlighted in the figure. The OAR sparing has been achieved for all the tested scenarios with no appreciable differences. The offsets lead to variations of target coverage for individual fractions, as shown in Figure 5. For all the offset scenarios, an underdosage was observed for several fractions. However, in the case of *σ*_1_ and *σ*_2_ the magnitude of that was small enough to be compensated by fractions in which the target coverage was even better than for no offset scenario. This is due to the random character of the simulated treatment execution uncertainties. Only for the largest offset corresponding to *σ*_3_, the variations were so large that the clinically acceptable level (*D*_98_ ≥ 95%) could not be achieved in the cumulative DVH. 

**Figure 6:**
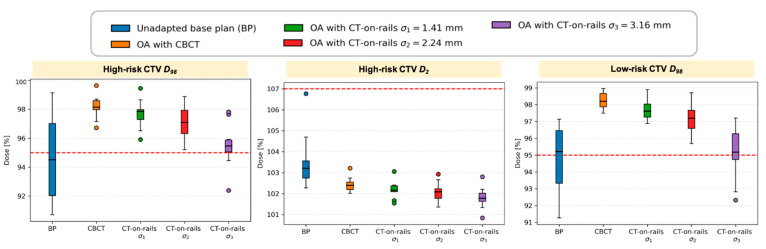
Summary of DVH metrics evaluated for the target in the whole patient cohort. Boxplots show: median (horizontal bar), Q1–Q3 (25th–75th) percentile (rectangle), 1.5 × (Q3–Q1) interquartile range (whiskers), outliers (dots).

The effect of the compromised target coverage (*D*_98_) for the scenario with *σ*_3_ was observed for several patients—2 for high-risk CTV and 5 for low-risk CTV, which can be seen in the boxplots summarizing the metrics evaluated for the target in the whole patient cohort (Figure 6). Although the spread of the *D*_98_ values was found to increase with increasing offsets, no single case with *σ*_1_ and *σ*_2_ failed to fulfill the clinical goal. The near-maximum dose *D*_2_ has decreased with increasing uncertainty *σ* and was well within the clinical goal. It is to be noted that online adaptation even with the largest treatment execution uncertainty *σ*_3_ still outperforms the unadapted base plan for the studied patient cohort.

**Figure 7:**
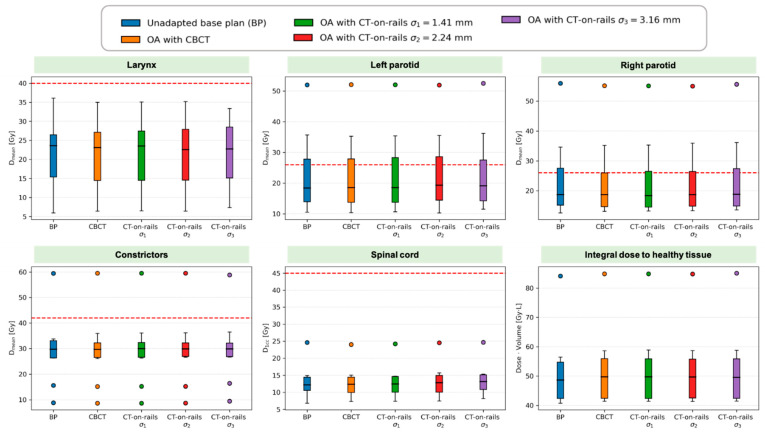
Summary of DVH metrics evaluated for OARs and healthy tissue in the whole patient cohort. Boxplots show: median (horizontal bar), Q1–Q3 (25th–75th) percentile (rectangle), 1.5 × (Q3–Q1) interquartile range (whiskers), outliers (dots).

For OARs, there were no significant differences observed between all the scenarios (Figure 7). Independent of the offset, for the parotid glands and constrictors, single outliers largely exceeded the mean dose corresponding to the clinical goal. This is because, for those cases, no constraint was applied to parotid glands (2 patients) and to constrictors (1 patient) in the treatment plan optimization due to the proximity of those organs to the target. 

The summary of the evaluated metrics in the whole patient cohort for all the regions of interest is presented in Table 2. For CTVs, the values not meeting the clinical goal are highlighted in red.

Table 3 presents the results of the comparison of DVH metric values obtained for three different CT-on-rails scenarios compared with those corresponding to CBCT-based online adaptation. *p*-values are reported, and statistically significant differences are highlighted. All the target metrics for the largest-offset (*σ*_3_) scenario are significantly different from those obtained with CBCT. For the intermediate-offset (*σ*_2_) scenario, the difference is significant only for low-risk CTV. However, as shown above, even though the absolute values are significantly different, all the cases meet the predefined clinical goals. 

## 4. Discussion

In this paper, we consider two imaging modalities for future adaptive treatments of head-and-neck cancers with protons. Two aspects represent the most important differences between CT-on-rails and CBCT for adaptive proton therapy—image quality and residual positioning uncertainties. In terms of dosimetric efficacy of daily adaptation, the image quality might affect dose calculation accuracy. That aspect has already been addressed in several studies focused on CBCT for daily adaptive proton therapy [22,23,24,25,26], proving the feasibility of accurate dose calculation on scatter-corrected CBCT images. Therefore, the difference in residual positioning uncertainties, due to the necessity of moving the patient after imaging when CT-on-rails is employed, becomes the primary factor that might compromise adaptive proton treatments. We conducted the first quantitative study on a cohort of head-and-neck patients to assess how much those uncertainties may affect the adaptation efficacy in terms of target coverage and accumulated dose distributions. 

The obtained results clearly indicate the extra uncertainties associated with the use of CT-on-rails do not affect dosimetric adaptation efficacy if the uncertainty due to couch-motion-induced patient’s movement *σ_m_* does not exceed 2 mm. This is a conservative limit, as we did not test scenarios with *σ_m_* laying between 2 and 3 mm. Moreover, the extra isocenter-matching uncertainty for CT-on-rails is conservatively large (*σ_i_* = 1 mm), as the aim for the total isocenter-matching uncertainty is usually below 1 mm, and CBCT iso-center matching, in reality, is also not perfect. We also assumed no movement of the patient on the couch between the in-treatment-position imaging with CBCT and dose delivery. Although it is less likely than in the case of CT-on-rails, the patient may also move during the treatment, at least in relation to the later fields of delivery.

The available data on patient position uncertainty while using CT-on-rails are scarce. Based on the white paper published by researchers from Paul Scherrer Institute (PSI) and Siemens Helthineers, the use of in-room CT for head-and-neck patients is associated with an accuracy of 0.3 mm and precision of 1.6 mm [29], which is well within the limit concluded from our study. 

Displacements from isocenter occurring during isocentric robotic rotations of the treatment couch have been recently reported in a study on the 3D surface imaging system AlignRT to verify patient positioning following couch rotations [38]. During isocentric rotations, the mean displacement AlignRT vectors for the phantom, immobilized, and non-immobilized volunteers were found to be 0.1 ± 0.1 mm, 0.8 ± 0.1 mm, and 1.1 ± 0.2 mm respectively. 

Daily imaging and positioning of patients outside of the treatment room, so-called remote positioning, is much less common than utilizing in-room CT. The proton therapy center at PSI has been using such a technique for over 20 years and performed a study on positioning errors associated with such an approach by acquiring pre- and post-treatment images [28]. For head-and-neck cancers, mean changes in patient position were found to be substantially less than 1 mm with standard deviations up to 1.7 mm for bite-block and 0.36–1.41 mm with standard deviations up to 2.4 mm for thermoplastic mask. The latter case exceeds the limit of 2 mm, and thus the efficacy of the adaptive treatments might be affected. However, the overall benefit of adaptation with respect to the use of an unadapted plan is still preserved. Therefore, in proton therapy centers that are equipped neither with in-room CT nor in-room CBCT, an adaptive approach based on remote positioning can be introduced, bringing improvement in dose conformality. 

The lack of datasets containing daily images of the same patient taken with both CBCT and CT-on-rails impose certain limits on the comparison between the two modalities. First, it is not possible to study the interplay between residual positioning uncertainties and range uncertainties affecting dose calculation accuracy. In fact, potentially lower range uncertainties for CT-on-rails due to weaker beam hardening and scatter might compensate larger treatment execution uncertainties leading to even better dosimetric efficacy than shown in this paper. Another consequence of using CBCT data only is the assumption that adaptation on vCTs is equivalent to adaptation on CBCT images which neglects DIR uncertainties. However, in the study focused on residual positioning uncertainties only, that assumption is justified as it allows relative dosimetric comparison to be made.

## 5. Conclusions

We have shown that CT-on-rails can be effectively used for daily adaptive proton therapy of head-and-neck cancers if the standard deviation of random patient movements induced by couch motion does not exceed 2 mm. Based on available data, the uncertainty for in-room CT will be lower than the concluded limit. Given other advantages of fan-beam CT, such as better image quality and potentially lower whole-body dose with respect to CBCT, CT-on-rails can be considered an excellent alternative to CBCT for adaptive proton therapy. 

## Figures and Tables

**Table 1:** Summary of major differences between imaging with CBCT and CT-on-rails.

Aspect	CBCT	CT-on-Rails
Image quality and dose calculation	Low image quality (scatter correction needed); contouring difficult Discrepancy in HU between planning CT and daily CBCT Potentially higher range uncertainties	High image quality; suitable for contouring Single modality for both planning and adaptation
Extra whole-body dose (based on [27])	1.11–4.95 mSv	0.8–2.38 mSv (excluding 4DCT)
Positioning uncertainty after daily adaptation	Negligible	Additional isocenter matching uncertainty σ_i_: - In-room CT Robot precision—σ_i_ < 1 mm - Remote positioning Coupling system—σ_i_ < 1 mm [28] Random displacement of the patient induced by couch motion σ_m:_ - Patient-specific - Tumor-site specific - σ_m_~1–3 mm [28,29]

**Table 2:** Median (min-max) values for DVH metrics evaluated for all regions of interest (ROI). For CTVs, the values not meeting the clinical goal are highlighted in red.

ROI	DVH Metric	Clin. Goal	Base Plan (BP)	OA with CBCT (No Offset)	OA with CT-on-Rails		
					*σ*_1_	*σ*_2_	*σ*_3_
High-risk CTV	*D*_98_ (%) *D*_2_ (%)	≥95 ≤107	94.5 (90.7–99.1) 103.2 (102.3–106.8)	98.1 (96.7–99.6) 102.4 (102.0–103.2)	97.8 (95.9–99.5) 102.2 (101.6–103.1)	97.1 (95.2–98.9) 102.1 (101.4–102.9)	95.5 (92.4–97.8) 101.8 (100.8–102.8)
Low-risk CTV	*D*_98_ (%)	≥95	95.2 (91.3–97.1)	98.2 (96.7–99.6)	97.6 (95.9–99.4)	97.2 (95.7–98.7)	95.2 (92.3–97.2)
Larynx	*D_mean_* (Gy)	<40	23.6 (5.9–36.1)	23.1 (6.4–35.0)	23.5 (6.5–35.1)	22.6 (6.4–35.2)	22.7 (7.3–33.4)
Right parotid	*D_mean_* (Gy)	<26 *	18.7 (12.5–56.0)	18.7 (13.1–55.2)	18.4 (13.2–55.1)	18.7 (13.3–55.0)	18.8 (13.6–55.6)
Left parotid	*D_mean_* (Gy)	<26 *	18.4 (10.6–52.0)	18.6 (10.4–52.1)	18.6 (10.7–52.1)	19.3 (10.3–52.0)	19.2 (11.6–52.6)
Constrictors	*D_mean_* (Gy)	<42 **	29.7 (8.9–59.5)	29.7 (8.6–59.6)	29.9 (8.6–59.6)	29.9 (8.7–59.5)	29.8 (9.4–58.9)
Spinal cord	*D*_1cc_ (Gy)	<45	12.2 (6.8–24.6)	12.4 (7.3–24.0)	12.5 (7.3–24.2)	12.9 (7.4–24.5)	13.1 (8.2–24.7)
Healthy tissue	*D V* (Gy L)	-	48.7 (40.7–84.1)	49.8 (41.4–84.9)	49.7 (41.4–84.9)	49.7 (41.4–84.8)	49.6 (41.5–85.1)

* No constraint applied for 2 patients due to the proximity to CTVs; ** No constraint applied for 1 patient due to the proximity to CTVs.

**Table 3:** *p*-values for the DVH metric values for three different CT-on-rails scenarios compared with CBCT-based online adaptation. Statistically significant differences (*p* ≤ .05) are highlighted in red.

ROI	DVH Metric	OA with CT-on-Rails		
		*σ*_1_	*σ*_2_	*σ*_3_
High-risk CTV	*D*_98_ (%) *D*_2_ (%)	.12 .19	.08 .08	<.001 .006
Low-risk CTV	*D*_98_ (%)	.06	.006	<.001
Larynx	*D_mean_* (Gy)	.72	.93	.86
Right parotid	*D_mean_* (Gy)	.97	.85	.68
Left parotid	*D_mean_* (Gy)	.97	.85	.73
Constrictors	*D_mean_* (Gy)	.79	.91	.97
Spinal cord	*D_1cc_* (Gy)	.91	.68	.57
Healthy tissue	*D V* (Gy L)	.85	.91	.97

## Data Availability

The data are not publicly available due to privacy restrictions.
